# Two cases of Ramsay-Hunt syndrome following varicella zoster viral meningitis in young immunocompetent men: case reports

**DOI:** 10.1186/s12883-023-03074-0

**Published:** 2023-01-27

**Authors:** Yun Su Hwang, Young Seo Kim, Byoung-Soo Shin, Hyun Goo Kang

**Affiliations:** 1 Department of Neurology, Jeonbuk National University Medical School and Hospital, Jeonju, South Korea; 2 Institute of Clinical Medicine of Jeonbuk National University, Biomedical Research Institute of Jeonbuk National University Hospital, Jeonju, South Korea; 3 Department of Neurology, Wonkwang University Medical School and Hospital, Iksan, South Korea

**Keywords:** Facial palsy, Ramsay-Hunt syndrome, Varicella Zoster Virus, Viral meningitis

## Abstract

**Background:**

Ramsay-Hunt syndrome (RHS) due to varicella zoster virus (VZV) infection is commonly reported in individuals aged at least 50 years or immunocompromised individuals. VZV infection may invade the central nervous system (CNS) and cause meningitis or encephalitis, which are more likely to occur in patients with chronic diseases such as diabetes and chronic renal failure. However, cases with VZV-induced concurrent RHS and CNS infections are rare.

**Case presentation:**

Two young male patients, aged 32 and 43 years, with no underlying disease developed VZV meningitis, followed by RHS involving cranial nerves VII and VIII. Both patients presented with symptoms of peripheral facial palsy, and dizziness accompanied by tinnitus and hearing loss, which appeared several days after the onset of fever and headache. These symptoms were documented as facial neuropathy and sensorineural hearing loss in the electrophysiologic studies. Lymphocyte-dominant pleocytosis and VZV positivity were confirmed from cerebrospinal fluid examination and polymerase chain reaction, respectively. The patients were treated with intravenous acyclovir and oral steroids simultaneously. Following the treatment completion, both patients were relieved of their headaches and fever; however, facial palsy, dizziness, and tinnitus persisted. They were followed up at the outpatient clinic.

**Conclusion:**

These cases confirmed that RHS and CNS infections can co-exist even in young adults with normal immune function and more importantly, that CNS infection can precede RHS. Since early detection and treatment of RHS improve the prognosis, it is critical to closely monitor patients with VZV meningitis or encephalitis considering the possible superimposition of RHS.

## Background

Varicella zoster virus (VZV) causes various symptoms when it is reactivated after a latent period following primary infection [1]. Common symptoms of VZV infection include painful skin rashes and trigeminal ganglio-neuritis [2]. Additionally, Ramsay-Hunt syndrome (RHS) refers to a disease caused by VZV that typically presents with facial palsy and vesicles in the external auditory canal and is characterized by symptoms such as external ear pain, hypoacusis due to multiple cranial nerve disorders, tinnitus, and dizziness [3, 4]. RHS frequently affects elderly or immunosuppressed individuals [5, 6].

Further, VZV infection may invade the central nervous system (CNS) and present as isolated clinical meningitis, which refers to a condition that presents with leukocytosis in cerebrospinal fluid (CSF) accompanied by more than two of the following symptoms: headache, nausea, and vomiting, sensitivity to light, neck stiffness and fever [1, 7]. Although a few studies have reported that VZV infrequently infects the CNS, it is a significantly common source of CNS infection [8, 9]. Comorbidities (e.g., diabetes or chronic renal failure) may lead to an aggressive course or predispose patients to encephalitis or meningitis upon VZV infection. However, VZV rarely causes concurrent RHS and CNS infections [4]. The few such reported cases are limited to children and elderly patients (60 years or older) with chronic disease [4–6].

We report two such rare cases of concurrent VZV meningitis and RHS in two young men without any underlying diseases.

### Case presentation

#### Patient 1

A 32-year-old man presented to the emergency room with a persisting headache for seven days. The headache had visual analog scale (VAS) score of 3 on the first day of onset, and its severity gradually increased to VAS score of 7 on the day of admission. On the 4th day of onset, he developed an intermittent fever. Concurrently, a small vesicular lesion erupted around the right ear with mild pain, suggestive of shingles in the ear. At the time of admission, the patient expressed severe nausea in addition to the above symptoms, and the following vital signs were recorded: blood pressure of 125/87 mmHg, pulse rate of 93 beats/min, the respiration rate of 18 breaths/min, and body temperature of 37.7 °C. He reported a history of chickenpox when he was young. He had no underlying medical condition and was not taking any medication.

Neurological examination revealed subtle stiffness of the neck, and a positive result was confirmed in the jolt accentuation test. The hematological tests revealed elevated C-reactive protein (CRP) at 18.4 mg/L (normal range: 0-5 mg/L), whereas the other blood tests including complete blood cell count (CBC), erythrocyte sedimentation rate (ESR), liver and renal function tests, and electrolytes were within the normal range. Brain magnetic resonance image (MRI) with contrast yielded normal findings. CSF examination revealed a slightly increased intracranial pressure (210 mmH_2_O) and CSF protein (255.5 mg/dL), lymphocyte-dominant pleocytosis (800/mm^3^, lymphocytes 90%), and a slightly decreased CSF/serum glucose ratio (47/105). Polymerase chain reaction (PCR) test of the CSF confirmed VZV positivity. Bacteria, fungi, and tuberculosis were neither detected in cultures, nor upon staining of the serum and CSF. Serologic antibody tests for various infections such as syphilis, tuberculosis, human immunodeficiency virus, and others were negative and the chest x-ray revealed no tuberculous lesion.

**Fig. 1:**
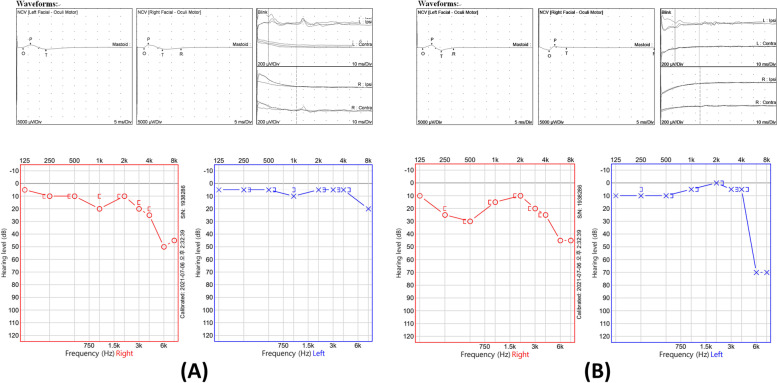
Facial NCS, blink reflex tests, and pure tone audiometry of patients 1 and 2. In both patients, the blink reflex test reveals absent responses of ipsilateral R1 and R2 and contralateral R2 at stimulation of the right supraorbital nerve. Moreover, pure tone audiometry reveals both increased air and bone conduction thresholds without an air–bone gap in the right ears. In patient 1 (**A**), facial NCS reveals nonspecific findings, and in patient 2 (**B**), facial NCS reveals prolonged terminal latency and reduced compound muscle action potential amplitude at direct stimulation of the right facial nerve. These findings suggest right-sided facial neuropathy and sensorineural hearing loss in both patients. NCS, nerve conduction studies

Despite the high CSF protein level, other laboratory CSF findings indicated viral meningitis alone with no other evidence of bacterial, fungal, or tuberculous infection in CSF, serum, and brain MRI, except for VZV positivity and a prior history of chickenpox. Consequently, the patient was administered intravenous acyclovir 10 mg/kg every 8 h considering the possibility of VZV meningitis. Antipyretics and analgesics were used to control fever, headache, and pain related to the vesicular lesions around the ear. On the third day of hospitalization, he developed a peripheral-type of facial palsy. Moreover, on the eleventh day of hospitalization, symptoms progressed to the severe spinning type of vertigo and tinnitus, with spontaneous left-beating nystagmus corresponding to Alexander's law. The symptoms corresponded to right-sided facial neuropathy considering that ipsilateral R1, R2, and contralateral R2 responses were not observed upon the stimulation of the right supraorbital nerve in the subsequent blink reflex test (Fig. 1A). Pure tone audiometry revealed increased thresholds of bone and air conduction in frequencies exceeding 2000 Hz in the right ear, which suggested high frequency-specific sensorineural hearing loss (Fig. 1A). Oral steroid (50 mg of prednisolone for 7 days followed by gradual tapering) was administered to treat RHS accompanying VZV meningitis. Antiviral injections were administered for a total of 14 days from the day of admission. At the time of discharge (20th day of hospitalization), the patient's headache and fever had subsided significantly. However, he is currently under follow-up at the outpatient clinic for persisting dizziness, tinnitus, and facial palsy.

### Patient 2

A 43-year-old man presented to the emergency room complaining of a headache from past 10 days, and right-sided facial palsy that had developed six days after headache onset and persisted thereafter. It was a throbbing type of headache accompanied by pain in the right ear. The patient had received seven days of antiviral and steroid treatment at another hospital due to suspected shingles, since the third day of the headache onset. At the time of admission to our hospital, the patient complained of a headache, which had slightly reduced compared to its first occurrence, along with right facial palsy, hypogeusia, vesicles and pain around the right ear, and hearing loss accompanied by dizziness and tinnitus. The following vital signs were recorded: blood pressure, 140/100 mmHg; pulse rate, 98/min; respiration rate, 20/min; and body temperature, 37.9 °C. He neither reported any underlying medical conditions nor was he under any medication.

**Fig. 2:**
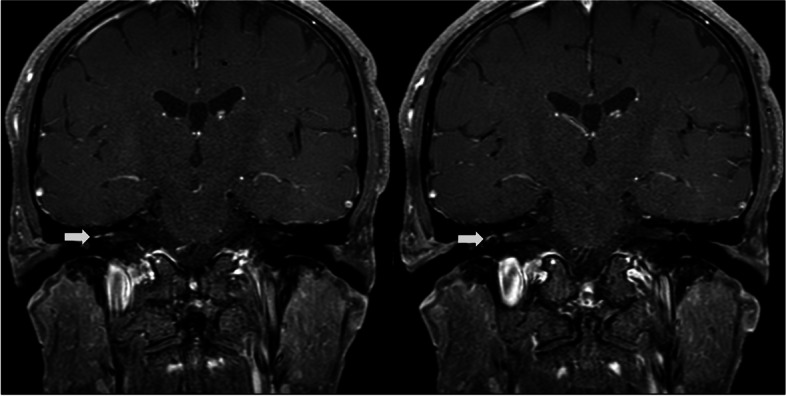
Brain magnetic resonance image of patient 2. Contrast-enhanced T1-weighted image reveals enhancement in the right facial nerve (White arrows)

Neurological examination revealed no abnormalities except for right-sided peripheral facial palsy. Blood tests including CBC, ESR, CRP, liver and renal function tests, and electrolytes revealed no specific findings. An abnormal enhancing lesion was observed in the right facial nerve on a brain MRI with contrast, suggestive of right facial neuritis (Fig. 2). CSF examination revealed an intracranial pressure of 80 mmH_2_O, lymphocyte-dominant pleocytosis (53/mm^3^, lymphocytes 98%), increased CSF protein (61.0 mg/dL), and a CSF/serum glucose ratio of 70/117. A CSF PCR test confirmed VZV positivity. Bacteria, Mycobacterium tuberculosis or fungi were not detected in cultures, nor on staining for serum and CSF. A facial nerve conduction study revealed prolonged terminal latency and decreased CMAP amplitude of the right facial nerve upon its direct stimulation. Moreover, no ipsilateral R1, R2, and contralateral R2 responses were observed upon stimulation of the right supraorbital nerve in a blink reflex test. The overall findings were indicative of right-sided facial neuropathy (Fig. 1B). Pure tone audiometry revealed increased bone and air conduction thresholds in frequency ranges approximating 500 Hz, and exceeding 2000 Hz on the right side, suggesting sensorineural hearing loss (Fig. 1B).

Medication included 10 mg/kg intravenous acyclovir every eight hours to treat VZV meningitis and oral steroid administration to treat facial palsy for seven days considering the prior history of already seven days of antiviral treatment in the other hospital before visiting our clinic. The accompanying pain was treated symptomatically. At the time of discharge, the patient's headache and dizziness had subsided, and the remaining facial palsy, auditory symptoms, and pain around the ears were treated symptomatically. He is currently on follow-up at the outpatient clinic for the residual symptoms.

## Discussion and conclusion

This study reported two cases of RHS after the onset of VZV meningitis in young men with normal immunity and no underlying diseases such as an immunocompromised state, diabetes, or chronic renal failure. RHS causes facial nerve palsy as VZV infection invades the VII cranial nerve and is also characterized by painful vesicles in the external auditory canal.^3,10^ It may also be associated with multiple neurological symptoms such as hearing impairment, tinnitus, and vertigo, often by invading the VIII cranial nerve, or rarely the IX, X, XI, and XII cranial nerves [3, 10]. CNS infection due to VZV presents as encephalitis, meningitis, vasculopathy, and myelitis with or without blisters [7, 11]. However, RHS and VZV meningitis rarely occur concurrently [4]. The two current cases without any underlying predisposing factor emphasize that VZV infection might frequently occur irrespective of the presence of any underlying disease or immunosuppressions similar to findings of previous research on CNS infection caused by VZV [8, 12].

As per our knowledge, previous concurrent RHS and CNS infection cases due to VZV involved either elderly patients, infants, or with chronic illnesses [4–6], or RHS cases arising from meningoencephalitis with decreased consciousness or focal neurological symptoms, but not isolated clinical meningitis [2, 12]. Furthermore, RHS in meningoencephalitis has also been reported in patients with underlying medical conditions [2]. Another study identified four RHS patients among 14 meningoencephalitis patient (mean age of 71.5 years) [12]. The patient 1 in this study developed facial palsy a few days after the diagnosis of meningitis, and involvement of the VIII cranial nerve was also confirmed through dizziness and tinnitus symptoms. It was presumed that VZV was reactivated following a latent period after the primary infection, given that this patient had a history of chickenpox. Patient 2 in this study exhibited concurrent facial palsy and auditory symptoms, suggesting VII and VIII cranial neuropathies from the time of hospital admission. However, we believe that meningitis occurred first as he had a headache and fever six days prior to the onset of RHS.

In both of our patients, VZV meningitis was diagnosed first and thereafter RHS symptoms occurred, therefore, the steroid was administered to the patients in addition to antiviral agents, which are similar in treatment to two previous cases [5, 6]. The use of steroid requires caution in viral meningitis due to the immunosuppressive effect, but based on the reducing effect of steroids on the inflammatory process in CNS infection [13] and those previous cases [5, 6], anti-inflammatory treatment for RHS were implemented in our cases. However, in these rare cases with concurrent RHS and CNS infection, there is not enough evidence for use of steroid, so careful determination should be taken for use of steroid [13].

Unfortunately, the mechanism of progression of CNS infection to RHS in both the patients of this study remains unclear. CSF infection can be a source of cochlear deficit, which indicates to a cranial nerve VIII dysfunction [4].Therefore, if auditory symptoms or dizziness (symptoms of cranial nerve VIII palsy) occur before the onset of facial palsy (a symptom of cranial nerve VII palsy), we hypothesize that the CSF infection invades the VIII cranial nerve first [4], and thereafter spreads to VII cranial nerve because of the close proximity between the VIII cranial nerve and the geniculate ganglion [10]. However, patient 1 experienced facial palsy first, followed by dizziness and tinnitus a few days later. Patient 2 reported an unclear temporal sequence between the invasion of cranial nerve VII and that of cranial nerve VIII. Another possible mechanism, in addition to the direct spread of CNS infection into the cranial nerves, is that VZV induces CNS infection after intracranial invasion through retrograde axonal spread from the geniculate ganglia, and then causes RHS through downward spread [4, 5]. The exact route of infection should be identified though more reports and experimental investigations.

RHS invading multiple cranial nerves is treated by medication therapy including combined antiviral agents and steroids administration, similar to Bell's palsy; additionally, facial palsy in RHS has a worse prognosis than Bell's palsy, wherein sequalae remain in severe cases due to incomplete recovery [10]. The precise prognosis for multiple cranial nerve disorders other than facial nerve palsy has not been established. However, prompt and accurate diagnosis is critical because early initiation of steroid and antiviral combination therapy can improve the prognosis by increasing the possibility of complete recovery from the sequelae [5, 14].

The cases in this study confirmed that RHS and CNS infection can concurrently develop even in young adults who are not predisposed to immunosuppression. They particularly showed that the clinical symptoms of RHS may develop in addition to symptoms of meningitis that have already manifested. Immediate treatment initiation after the early detection of RHS improves the prognosis. Therefore, it is critical to closely monitor patients with VZV meningitis or encephalitis when considering possible concurrent RHS.

## Data Availability

All data and material supporting our findings are contained within the manuscript.

## Abbreviations

CBC: Complete blood cell count
CNS: Central nervous system
CRP: C-reactive protein
CSF: Cerebrospinal fluid
ESR: Erythrocyte sedimentation rate
MRI: Magnetic resonance image
NCS: Nerve conduction study
PCR: Polymerase chain reaction
RHS: Ramsay-Hunt syndrome
VAS: Visual analog scale
VZV: Varicella zoster virus
